# The association between habitual physical activity and cigarette cravings, and influence of smokers’ characteristics in disadvantaged smokers not ready to quit

**DOI:** 10.1007/s00213-016-4326-6

**Published:** 2016-06-02

**Authors:** M. Haasova, F. C. Warren, T. Thompson, M. Ussher, A. H. Taylor

**Affiliations:** University of Exeter Medical School, St. Luke’s Campus, Heavitree Road, Exeter, EX1 2LU UK; Plymouth University Peninsula Schools of Medicine and Dentistry, Plymouth Science Park, Plymouth, PL6 8BX UK; St George’s University of London, Cranmer Terrace, London, SW17 0RE UK

**Keywords:** Exercise, Urges to smoke, Cigarette cravings, Cross-sectional, Disadvantaged, Smoking, Alcohol

## Abstract

**Rationale:**

Habitual physical activity (PA) may have an important role in suppressing cigarette cravings. Systematic reviews show a strong acute effect of bouts of PA on reducing cigarette cravings, and it may be that these effects accumulate.

**Objectives:**

The aim was to investigate the relationship between habitual levels of PA and cigarette cravings in disadvantaged smokers not ready to quit by examining baseline cross-sectional data from the Exercise Assisted Reduction then Stop smoking study (EARS).

**Methods:**

A series of linear regression models were applied to investigate the relationship between habitual PA and cigarette cravings and to identify additional predictors of cigarette cravings. The analyses were extended by including interaction terms with PA to identify potential moderators of the relationship between PA and cravings.

**Results:**

A higher level of moderate intensity PA was associated with lower cravings (*p* = 0.033). Additional predictors were the mood and physical symptoms scale (*p* = 0.007; higher scores were associated with higher cravings) and alcohol consumption (*p* = 0.002; higher consumption was associated with lower cravings). In addition, a moderation effect of alcohol consumption was found; at higher levels of alcohol consumption, higher PA was significantly associated with higher cravings (*p* = 0.023).

**Conclusions:**

Overall, participation in regular PA is associated with reduced cigarette cravings; among those with heavy alcohol consumption, this participation is associated with higher cravings. These exploratory analyses suggest that further research into the relationship between PA, alcohol consumption and cigarette cravings is needed.

**Electronic supplementary material:**

The online version of this article (doi:10.1007/s00213-016-4326-6) contains supplementary material, which is available to authorized users.

## Introduction

There is evidence that smokers are less physically active than non-smokers (Kaczynski et al. 2008) and that active smokers are more likely to quit smoking (Marcus et al. 2005). Cravings for cigarettes are a reliable predictor of relapse to smoking (e.g. Killen and Fortmann 1997; Piasecki et al. 2003), brief bouts of physical activity (PA) have a strong acute effect on reducing cigarette cravings (Haasova et al. 2013; 2014; Roberts et al. 2012), and it may be that these effects accumulate across the day. For example, Bloom et al. (2012) reported a negative association between cigarette withdrawal symptoms and weekly PA but only among male psychiatrically hospitalised adolescents, and not females (Bloom et al. 2012). It is important to determine if habitual or chronic PA is associated with both acute and more enduring or stable cravings (Tiffany and Wray 2012) in the general population of smokers and to examine whether these associations vary according to different sub-groups. Such evidence can inform the design of PA interventions for smoking cessation (Ussher et al. 2014).

There may be several factors that influence the strength of the relationship between PA and cravings, such as exercise characteristics (e.g. moderate and vigorous exercise has greater acute effects than light intensity; Haasova et al. 2014), socio-demographic factors (e.g. gender differences in motivation for smoking and PA; Bloom et al. 2012), satisfaction from smoking and cigarette dependence. There are also a number of shared correlates of smoking and PA, with lower socio-economic groups more likely to smoke, drink alcohol and being less active (Bellis et al. 2016). The relationship between PA and alcohol is not simple. Less physically active are more likely to consume alcohol (e.g.Korhonen et al. 2009; Liangpunsakul et al. 2010); however, other studies found the reverse association between PA and drinking (e.g. French et al. 2009a; Kwan et al. 2014; Lisha and Sussman 2010). Mood and measures of emotional well-being are more positive in response to PA (Reed and Ones 2006), and so it is important to control for these factors when assessing the association between PA and cravings. Exploratory work is therefore needed to tease out what factors may moderate the relationship between PA and cravings.

The aim of this study was to investigate the relationship between habitual levels of PA and cigarette cravings in disadvantaged smokers not ready to quit by examining baseline cross-sectional data collected in the pilot randomised trial Exercise Assisted Reduction then Stop smoking study (EARS; Taylor et al. 2014). Additional predictors of cigarette cravings, and potential moderators of the association between habitual levels of PA and cigarette cravings, were also explored.

## Methods

The EARS smoking study (Taylor et al. 2014) was a pragmatic, two-arm pilot randomised controlled trial comparing counselling on PA and smoking reduction (to cut down, then quit), with brief advice on quitting, among disadvantaged smokers who did not wish to quit in the next month. This study examines baseline data for all EARS participants, pooling intervention and control arm baseline data.

### Participants

Eligible participants were as follows: at least 18 years old, smoked at least 10 cigarettes per day for at least 2 years, did not want to quit in the next month, were able to walk continuously for at least 15 min, were registered with a general physician and did not wish to use nicotine replacement therapy to reduce smoking. The EARS study recruited 99 participants between May 2011 and May 2012.

### Measures

Only measures considered in this study are described below; a full list of outcomes is provided elsewhere (Taylor et al. 2014).

#### Physical activity

Self-reported PA was assessed by 7-day recall (Blair et al. 1985). Average nightly sleep and minutes of moderate and vigorous intensity PA over the past 7 days were recorded, and minutes of light activity were derived, with light intensity PA including any sedentary time. Minutes of moderate and vigorous intensity PA were then combined (MVPA), and participants were categorised as meeting the national PA guidelines (150 or more minutes of MVPA per week) or not meeting the guidelines (Bull and the Expert Working Groups 2010). In addition, daily energy expenditure (EE; kcal/kg) was calculated from self-reported PA (Blair et al. 1985).

#### Cigarette cravings

A 0–5-point Likert scale assessed strength of urge to smoke (SOU; 0 = no urges to 5 = extremely strong urges): ‘How strong have the urges been to smoke this past week?’ (West and Hajek 2004; West and Russell 1985).

#### Smoking measures

Self-reported numbers of cigarettes smoked over the past week, smoking satisfaction and psychological reward (10-item modified Cigarette Evaluation Questionnaire [mCEQ]; Cappelleri et al. 2007), cigarette dependence (Fagerström Test for Cigarette Dependence [FTCD]; Fagerström 2012; Heatherton et al. 1991) and the age when participants started smoking were collected.

#### Withdrawal symptoms

The mood and physical symptoms scale (MPSS) with nine items (restless, irritable, depressed, hungry, poor concentration, poor sleep at night, stressed out and tense) and 1–5-point Likert response range (‘not at all’ to ‘extremely so’) were used (West and Hajek 2004).

#### Quality of life

Data was collected on health-related quality of life using the three-level European Quality of Life-5 Dimension questionnaire (EQ-5D-3L; Prieto and Sacristan 2004). In addition, a binary variable ‘presence of a mental health condition’ indicating the presence/absence of mental health issues was derived from the EQ-5D-3L data. An answer indicating ‘some problems’ or ‘extreme problems’ on the anxiety/depression dimension was considered as an indication of potential mental health issues.

#### Subjective stress

The 4-item perceived stress scale (PSS) measured the extent to which respondents have felt their life to be stressful during the past month using a 5-point Likert scale (Cohen et al. 1983).

#### Alcohol consumption

Alcohol consumption was assessed using modified questions from the Alcohol Use Disorders Identification Test (AUDIT; Allen et al. 1997): (1) How often do you have a drink containing alcohol?; (2) How many drinks containing alcohol do you have on a typical day when you are drinking?; and (3) How many drinks containing alcohol have you had in the past week? The last two questions were answered only by participants who reported that they drink alcohol at least once a month or more frequently (*N* = 84; see Online Resource 1 for more details). Based on preliminary analyses, the first two AUDIT questions were combined into a new three-level alcohol consumption variable (*N* = 99; non-drinkers, light/moderate drinkers [drinking 1–6 drinks on a typical day drinking] and heavy drinkers [drinking 7 or more drinks on a typical day drinking]) and used in the analyses.

### Statistical analyses

Data were described using the mean and SD, median and inter-quartile range (IQR), and proportions. Associations were investigated using correlations and linear regressions models. To facilitate the use of linear regression and to assist with interpretation of the results, although technically ordinal variables, measures of cigarette cravings were linearly rescaled to a range of 0–100 and were treated as continuous variables (Lyratzopoulos et al. 2012). All statistical analyses were performed using Stata 13, and the significance threshold was set at 0.05 in all analyses. No attempts to impute missing data were made; consequently, some analyses did not include the whole dataset (*N* = 95–99) and, where there is missing data, this is indicated in the results. All analyses described in this section are post hoc exploratory analyses. In many analyses, multiple tests of the trial outcomes were performed. Therefore, all results must be interpreted with caution.

#### The association between physical activity and strength of urges to smoke

To investigate a potential association between habitual levels of PA and cigarette cravings, a series of linear regression models were applied individually with minutes of MVPA and total EE as predictors of SOU. The likelihood ratio (LR) test was used to compare the fit of nested models to assess whether the model including moderate intensity PA could be improved.

#### Additional predictors of strength of urges to smoke

The associations between potential predictors and SOU were investigated using a series of univariable linear regression models. Each univariable model was then repeated with adjustment for PA. All individually significant additional predictors (after adjusting for PA) identified in the individual regression models were combined in one model. All variables that were significantly associated with SOU in the individual regression models were also combined in a backward stepwise regression model. In addition, all individually significant variables were included in a backward stepwise regression model with the moderate intensity PA variable included as a predictor regardless of its contribution to the model. These models aimed to identify additional predictors of cigarette cravings, with and without adjustment for PA.

#### Moderators of the association between physical activity and strength of urge

The analyses of individually significant predictors of SOU (after adjusting for PA) were extended by including interaction terms with PA. Individual models (i.e. each including only one potential moderator) with the potential moderator, PA and potential moderator/PA interaction were applied to the data. Only variables demonstrating a significant interaction with PA were considered to be moderating the effects of habitual PA on cigarette cravings (Kraemer et al. 2002). If appropriate, all moderators identified in the individual regression models were combined in one model to identify all significant moderators of the relationship between PA and cravings. The LR test was used to compare the fit of nested models. We also investigated any potential moderation of the effects of PA by variables found not to be individually associated with cigarette cravings. Finally, in order to identify the most appropriate model, all significant predictors and moderators were combined. The LR test was used to compare the fit of nested models.

## Results

Participants’ characteristics are summarised in Table 1.

**Table 1:** Participants characteristics (*N* = 99)

Variables		Mean (SD); median [IQR] or *n*/*N* %
Strength of urge (0–5 scale)		2.7 (1.2); 3.0 [2, 3]
Minutes of light PA per day		992.4 (139.4); 1020 [908.6, 1088.6]
Minutes of moderate PA per day^a^		70.3 (88.5); 45 [17.1, 77.1]
Minutes of vigorous PA per day		2.8 (14.1); 0 [0, 0]
Minutes of MVPA per day^a^		73.1 (91.1); 45 [17.1, 77.1]
Daily energy expenditure (kcal/kg)^b^		36.05 (3.9); 34.9 [33.7, 36.8]
Age (years)		46.6 (11.3); 47.5 [38.3, 55.4]
EQ-5D-3L^c^		0.749 (0.275); 0.796 [0.725, 1]
PSS (0–16 score range)		5.7 (4.1); 4 [2, 9]
MPSS (1–5 score range)		2.5 (0.9); 2.3 [1.8, 3.1]
Cigarettes smoked per day		21.6 (14.3); 19.1 [14.4, 24.4]
FTCD (0–10 score range)		5.6 (2.0); 6.0 [4.0, 7.0]
Age started smoking (years)		14.7 (3.5); 14.0 [13.0, 16.0]
mCEQ satisfaction (1–7 score range)		3.8 (.5); 3.7 [2.7, 4.7]
mCEQ reward (1–7 score range)		3.3 (1.2); 3.2 [2.6, 4.2]
Alcohol consumption:^d^	Non-drinkers:	15/99 (15)
	Light/moderate drinkers:	60/99 (60)
	Heavy drinkers:	24/99 (24)
Gender male:		43/99 (43)
Presence of mental health condition^e^		41/99 (41)
Employed		54/99 (55)
Meeting PA guidelines^a, f^		68/98 (70)

Key: EQ-5D-3L, three-level European Quality of Life-5 Dimension questionnaire, *FTCD* Fagerström Test for Cigarette Dependence, *IQR* inter-quartile range, *mCEQ* modified Cigarette Evaluation Questionnaire, *MET* Metabolic equivalent of Task, *MPSS* mood and physical symptoms scale, *MVPA* Moderate and Vigorous Physical Activity, *N* Number of participants, *PSS* perceived stress scale, *SD* standard deviation

^a^ *N* = 98

^b^ *N* = 95

^c^Score of 1 represents full health, zero represents death, while negative score represents states worse than death

^d^Three-level ‘Alcohol consumption’ variable combined from the first two AUDIT questions;‘non-drinkers’ (no alcohol consumption), ‘light/moderate drinkers’ (consuming between 1 and 6 alcoholic drinks on a typical day) and ‘heavy drinkers’ (consumed 7 or more alcoholic drinks on a typical day). For the results of the three modified AUDIT questions see Online Resource 1

^e^Answered ‘moderately’ or ‘extremely’ anxious or depressed to item 5 of the EQ-5D-3L questionnaire

^f^Participants were categorised as meeting the PA guidelines (exercising 150 or more minutes of MVPA per week) and not meeting the PA guidelines (exercising less than 30 or more minutes of MVPA per week)

### The association between physical activity and strength of urge

Table 2 summarises the association between self-reported PA (minutes of daily light, moderate, vigorous and MVPA intensities and EE) and SOU. All levels of habitual PA, except vigorous intensity, predicted SOU (*p* < 0.05). Light intensity PA (including sedentary time) was positively associated with SOU, whereas daily EE, moderate and MVPA intensities were negatively associated with SOU. Therefore, for an increase in moderate PA of 30 min per day (within the range of the observed data), a mean reduction in SOU of around 2.5 points would be expected (based on results in Table 2). Because only eight participants reported exercising at vigorous intensity PA, the results for moderate and MVPA intensities were very similar.

**Table 2:** Strength of urge; results of series of linear regression models including each physical activity variable individually (*N* = 99)

PA	SOU; mean difference (95 % CI)	*F* statistics (*p*)	*R* ^2^ (%)
Minutes of light PA per day^a^	*0.053 (0.02; 0.09)*	*F*_*(1,93)*_ *= 9.80 (0.002)*	8.6
Minutes of moderate PA per day^b^	*−0.082 (−0.13; −0.03)*	*F*_*(1,96)*_ *= 10.04 (0.002)*	8.5
Minutes of vigorous PA per day	−0.054 (−0.39; 0.28)	F_(1,97)_ = 0.10 (0.746)	−0.9
Minutes of MVPA per day^b^	*−0.078 (−0.13; −0.03)*	*F*_*(1,96)*_ *= 9.79 (0.002)*	8.3
Daily energy expenditure^a^ (kcal/kg)	*−1.586 (−2.77; −0.40)*	*F*_*(1,93)*_ *= 7.09 (0.009)*	6.1

Statistically significant results are shown in italics

Key: *95 % CI* 95 % confidence interval, *MVPA* moderate and vigorous physical activity, *N* number of participants, *PA* physical activity, *R* ^*2*^ adjusted *R* ^2^, *SOU* strength of urge

^a^ *N* = 95

^b^ *N* = 98; strength of urge was linearly rescaled to 0–100 scale

We then investigated whether the addition of other PA variables to the univariate model including moderate intensity PA would improve the model fit (MVPA was not included because of collinearity with moderate intensity PA). Based on LR tests, the addition of neither light intensity PA nor EE to a model including moderate intensity PA alone significantly improved the model fit. Therefore, in all subsequent models, only moderate intensity PA was included as a measure of habitual PA.

### Additional predictors of strength of urge

Categorical variables (alcohol consumption, gender, employment, meeting PA guidelines, presence of a mental health condition), and continuous variables (EQ-5D-3L, MPSS, FTCD, mCEQ reward), significantly correlated with SOU, or approaching significance (PSS ([*p* = 0.064]; for the correlations see Online Resource 2) were included in series of linear regression models investigating the effects of each potential predictor individually on SOU. PSS, MPSS, FTCD, mCEQ reward, alcohol consumption and presence of a mental health condition were found to be individually significantly associated with SOU (see Online Resource 3). The same six variables, PSS, MPSS, FTCD, mCEQ reward, alcohol consumption, and presence of a mental health condition, remained significantly associated with SOU after adjusting for moderate intensity PA (Table 3). In addition, moderate intensity PA remained significant when included in all models with one additional significant variable.

**Table 3:** Series of linear regression models investigating the associations of each potential additional predictor individually on strength of urge (adjusted for moderate intensity physical activity, *N* = 98)

		Additional predictors (as specified in the first column)		Moderate PA	
Additional predictors		SOU, mean difference (95 % CI)	*t* statistics (*p*)	SOU, mean difference (95 % CI)	*t* statistics (*p*)
EQ-5D-3L		−10.23 (−26.87; 6.41)	−1.22 (0.225)	*−0.08 (−0.13; −0.02)*	*−2.89 (0.005)*
PSS		*1.09 (0.01; 2.18)*	*2.01 (0.048)*	*−0.08 (−0.13; −0.03)*	*−3.02 (0.003)*
MPSS		*8.16 (3.28; 13.04)*	*3.32 (0.009)*	*−0.08 (−0.12; −0.03)*	*−3.06 (0.003)*
FTCD		*3.21 (1.06; 5.36)*	*2.96 (0.004)*	*−0.07 (−0.12; −0.02)*	*−2.81 (0.006)*
mCEQ reward		*5.51 (1.98; 9.03)*	*3.10 (0.003)*	*−0.08 (−0.13; −0.03)*	*−3.29 (0.001)*
‘Alcohol consumption’^a^	Light/moderate drinkers	*−25.36 (−37.08; −13.62)*	Global statistic^b^: *F*_*(2,94)*_ *= 11.43, p < 0.001*	*−0.077 (−0.12; −0.03)*	*−3.22 (0.002)*
	Heavy drinkers	−10.19 (−23.51; 3.14)			
Employment status		5.89 (−3.40; 15.18)	1.26 (0.211)	*−0.07 (−0.13; −0.02)*	*−2.80 (0.006)*
Presence of a mental health condition^c^		*10.80 (1.691; 19.91)*	*2.32 (0.021)*	*−0.07 (−0.12; 0.02)*	*−2.77 (0.007)*
Met PA guidelines		2.36 (−8.91; 13.63)	0.42 (0.678)	*−0.09 (−0.15; −0.03)*	*−2.95 (0.004)*
Gender		1.49 (−7.75; 10.73)	0.32 (0.750)	*−0.08 (−0.13; −0.03)*	*−3.06 (0.003)*

Statistically significant results are shown in italics

Key: *EQ-5D-3L* three-level European Quality of Life-5 Dimension questionnaire, *FTCD* Fagerström Test for Cigarette Dependence, *mCEQ* modified Cigarette Evaluation Questionnaire, *MPSS* mood and physical symptoms scale, *N* number of participants, *PA* physical activity, *PSS* perceived stress scale, *SOU* strength of urge, *5 % CI* 95 % confidence interval

^a^‘Non-drinkers’ (no alcohol consumption), ‘light/moderate drinkers’ (consuming between 1 and 6 alcoholic drinks on a typical day) and ‘heavy drinkers’ (consumed 7 or more alcoholic drinks on a typical day)

^b^ *p* values for the global *F* statistic of the three levels alcohol consumption are derived from a Wald test

^c^Answered ‘moderately’ or ‘extremely’ anxious or depressed to item 5 of the EQ-5D-3L questionnaire; ‘not drinking alcohol’ was the baseline category for ‘alcohol consumption’; ‘male’ was the baseline category for gender; ‘employed’ was the baseline category for employment status; ‘not meeting PA guidelines’ was the baseline category for Met PA guidelines; ‘lack of anxiety’ was the baseline category for presence of a mental health condition

When all individually significant predictors (PSS, MPSS, FTCD, mCEQ reward, alcohol consumption, presence of a mental health condition) and moderate PA were included in the same regression model with SOU, only moderate intensity PA, MPSS and alcohol consumption remained significant with SOU (*p* < 0.05). When the backward stepwise regression model was applied (using the same variables), moderate intensity PA, MPSS and alcohol consumption were included in the model (Table 4). Repeating the backward stepwise regression model with enforced inclusion of moderate intensity PA resulted in the same model. In summary, MPSS and alcohol consumption were found to be additional predictors (adjusted for moderate intensity PA) of SOU in this population (Table 4). Both an increase in moderate intensity PA and higher alcohol consumption were associated with lower SOU; the decrease in SOU associated with alcohol consumption appeared to be driven by lower SOU among light/moderate drinkers compared with non-drinkers (mean difference −23.46, 95 % CI −34.66 to −12.26). A higher MPSS score was associated with higher SOU.

**Table 4:** Stepwise regression model showing additional predictors of strength of urge to smoke (*N* = 98)

		Mean difference (95% CI)	*t* statistics (*p*)
Moderate intensity PA (minutes per day)		*−0.07 (−0.11; −0.03)*	*−3.14 (0.002)*
Alcohol consumption^a^	Light/moderate drinkers	*−23.46 (−34.66; −12.26)*	Global statistic^b^: *F*_*(2,93)*_ *= 11.41, p < 0.001*
	Heavy drinkers	−8.07 (−20.81; 4.66)	
MPSS		*7.42 (2.97; 11.86)*	*3.31 (0.001)*
*F* statistic		*F*_*(4,93)*_ *= 12.45; p < 0.001*	
*R* ^2^		0.349	

Statistically significant results are shown in italics

Key: *95 % CI* 95 % confidence interval, *PA* physical activity

^a^‘Non-drinkers’ (no alcohol consumption), ‘light/moderate drinkers’ (consuming between 1 and 6 alcoholic drinks on a typical day) and ‘heavy drinkers’ (consumed 7 or more alcoholic drinks on a typical day); moderate PA, PSS, MPSS, FTCD, mCEQ reward, alcohol consumption and presence of a mental health condition were included in the regression model; repeating the backward stepwise regression model without the moderate intensity PA variable kept in resulted in the same model; ‘non-drinkers’ was the baseline category for alcohol consumption

^b^ *p* values for the global *F* statistic of the three levels alcohol consumption are derived from a Wald test

### Moderators of the effect of moderate intensity physical activity on strength of urge

Each potential moderator and its interaction with PA were included in an individual model with PA; gender, presence of a mental health condition, EQ-5D-3L, PSS, MPSS, cigarettes smoked per day, FTCD, age started smoking, mCEQ reward, mCEQ satisfaction, alcohol consumption, employment status and meeting PA guidelines were considered. Only significant moderators are reported. Alcohol consumption was found to moderate the relationship between moderate intensity PA and SOU. The interaction term between alcohol consumption and minutes of moderate PA (a continuous variable) was significantly different from zero (*F*_(2,92)_ = 5.51, *p* = 0.006) overall (Table 5).

**Table 5:** Linear regression showing the three levels alcohol consumption moderator of strength of urge (adjusted for physical activity, *N* = 98)

		Mean difference (95 % CI)	*t* statistics (*p*)
Moderate intensity PA (minutes per day)		−0.12 (−0.24; −0.01)	−2.13 (0.035)
Three levels alcohol consumption^a^	Light/moderate drinkers	−25.46 (−39.07; −11.85)	Global statistic^b^: *F*_(2,92)_ = 7.05, *p* = 0.001
	Heavy drinkers	−22.96 (−38.80; −7.11)	
PA/ three levels alcohol consumption	Interaction between moderate intensity PA and ‘light/moderate drinkers’	0.01 (−0.12; 0.14)	Global statistic^b^: *F*_(2,92)_ = 5.51, *p* = 0.006
	Interaction between moderate intensity PA and ‘heavy drinkers’	0.19 (0.04; 0.34)	
*F* statistic		*F*_(5,92)_ = 9.90; *p* < 0.001	
*R* ^2^		0.350	

Key: *95 % CI* 95 % confidence interval, *PA* physical activity

^a^‘Non-drinkers’ (no alcohol consumption), ‘light/moderate drinkers’ (consuming between 1 and 6 alcoholic drinks on a typical day) and ‘heavy drinkers’ (consumed 7 or more alcoholic drinks on a typical day)

^b^ *p* values for the global *F* statistic of the three levels alcohol consumption are derived from a Wald test; ‘non-drinkers’ was the baseline category for drinking alcohol

**Fig. 1:**
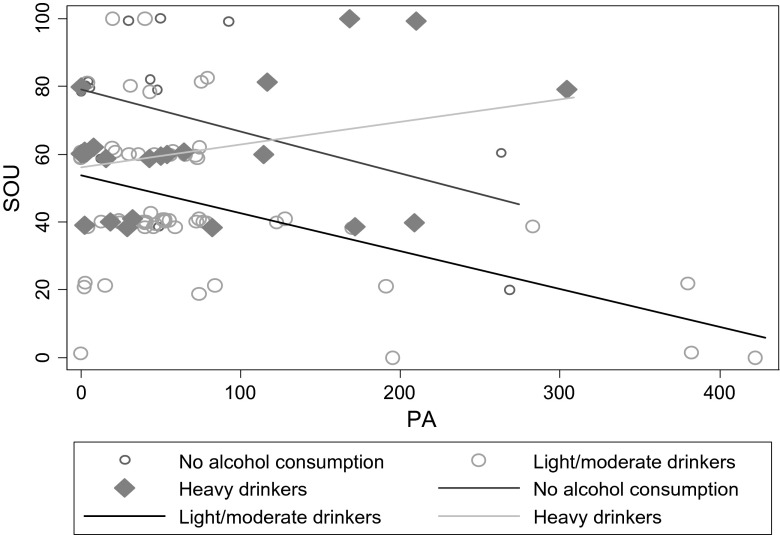
The relationship between Alcohol consumption and strength of urge in the whole population (*N* = 98). Notes: *PA* physical a, *SOU* strength of urge

It appears that the ‘non-drinkers’ subgroup reported higher cigarette cravings compared with ‘light/moderate drinkers’ and ‘heavy drinkers’; the global *F* statistic of the three levels alcohol consumption was statistically significant (Table 5). However, the moderating effects of alcohol consumption on the relationship between moderate intensity PA and SOU suggest that both the ‘non-drinkers’ and ‘light/moderate drinkers’ are associated with lower cigarette cravings as minutes of moderate PA increases, whereas ‘heavy drinkers’ are associated with higher cravings as minutes of moderate PA increases (Fig. 1).

### Combining additional predictors of strength of urge and moderators of the effect of moderate intensity physical activity on strength of urge

All significant predictors and moderators were analysed together to identify the most appropriate model. MPSS and alcohol consumption were identified as additional predictors of the effects of PA on SOU; alcohol consumption was also identified as a moderator of the effects of PA on SOU. The LR test was used to compare the fit of two models, the first including MPSS, PA, alcohol consumption and the interaction between PA and alcohol consumption and the second excluding the interaction term.

The LR test demonstrated that adding the interaction between PA and alcohol consumption improved the model; LR = 8.12, *p* = 0.0173. Thus, the most appropriate model predicting SOU in the whole population includes PA, MPSS, alcohol consumption, and the interaction between PA and alcohol consumption (Table 6); again, an increase in moderate intensity PA and higher alcohol consumption were associated with lower SOU, while a higher MPSS score was associated with higher SOU.

**Table 6:** Strength of urge; the most appropriate model in the whole population

		Mean difference (95 % CI)	*t* statistics (*p*)
Moderate intensity PA (minutes per day)		−0.12 (−0.23; −0.01)	−2.16 (0.033)
Three levels alcohol consumption^a^	Light/moderate drinkers	−24.64 (−37.77; −11.46)	Global statistic^b^: *F*_(2,91)_ = 6.91, *p* = 0.002
	Heavy drinkers	−19.53 (−35.05; −4.07)	
PA/ three levels alcohol consumption	Interaction between moderate intensity PA and ‘light/moderate drinkers’	0.02 (−0.10; 0.15)	Global statistic^b^: *F*_(2,91)_ = 3.93, *p* = 0.023
	Interaction between moderate intensity PA and ‘heavy drinkers’	0.67 (0.02; 0.31)	
MPSS		6.18 (1.76; 10.59)	2.78 (0.007)
*F* statistic		*F*_(6,91)_ = 10.13; *p* < 0.001	
*R* ^2^		0.309	

Key: *95 % CI* 95 % confidence interval, *PA* physical activity, *MPSS* mood and physical symptoms scale

^a^‘Non-drinkers’ (no alcohol consumption), ‘light/moderate drinkers’ (consuming between 1 and 6 alcoholic drinks on a typical day) and ‘heavy drinkers’ (consumed 7 or more alcoholic drinks on a typical day)

^b^ *p* values for the global *F* statistic of the three levels alcohol consumption are derived from a Wald test; ‘non-drinkers’ was the baseline category for alcohol consumption

## Discussion

This is the first study to indicate that habitual moderate intensity PA is associated with SOU over the past week. Higher levels of moderate intensity PA (measured as average minutes per week over the preceding week) were associated with lower SOU over the same period in disadvantaged smokers not ready to quit. Both the acute studies (Haasova et al. 2014) and the current study suggest that moderate intensity PA is the strongest PA predictor of cigarette cravings; however, it must be highlighted that the design and population of the studies varied significantly. The effects of habitual moderate PA appeared to be much smaller when compared with the acute data; an increase in habitual moderate PA of 5–40 min per day (within the range of the observed data) was associated with a mean reduction in SOU of around 0.4–3.3 points on a 0–100 scale compared with an approximate cravings reduction of 30 % associated with acute bouts of PA lasting from 5–40 min (Haasova et al. 2013; 2014). A recent Cochrane review of exercise interventions for smoking cessation identified 20 RCTs comparing exercise and cessation programmes with at least 6 months follow-up (Ussher et al. 2014). However, only one study (Marcus et al. 1999) showed long term benefits of exercise on smoking cessation. Notably, most studies included in the Cochrane review did not aim to increase levels of habitual PA (Ussher et al. 2014); regular participation in PA, rather than 1–3 sessions per week, may have greater enduring effects on smoking cessation outcomes.

MPSS and alcohol consumption were identified as two additional predictors of SOU; also, participants who consumed alcohol had lower cravings compared with non-drinkers. Higher alcohol consumption was associated with lower SOU, while a higher MPSS score was associated with higher SOU. The difference between participants who consumed alcohol and those who did not cannot be readily explained. Whilst the relationship between PA levels and smoking prevalence is quite distinct, the relationship between PA and alcohol is much more complicated and is rarely reported to be linear in nature as it is with smoking (French et al. 2009a; Lisha et al. 2013; Noble et al. 2015). Also, the relationship between PA and alcohol may be influenced by the type of activity (Wichstrom and Wichstrom 2009), so a complicated relationship and interactions are to be expected (Ekkekakis 2013; Ussher 2014). One possible explanation for the finding may be that participants who consumed alcohol reported lower cravings as their threshold for smoking was lower compared with participants who did not consume alcohol. Indeed, ‘non-drinkers’ reported higher cigarette cravings compared with ‘light/moderate drinkers’ and ‘heavy drinkers’ across all levels of PA. In addition, if an addiction is founded on reward and pleasure derived from a behaviour, then it is possible that in the present case heavy drinkers satisfy that need from drinking and hence have a lower need or urge for cigarettes. While an alternative scenario could be that high levels of alcohol consumption actually reduces inhibition to refrain from smoking when faced with high urges to smoke, our findings do not appear to support this.

Interestingly, measures of smoking dependence, such as FTCD and the number of cigarette smoked per day, were not found to predict SOU in addition to moderate habitual PA, MPSS and alcohol consumption. The association between habitual PA and trait cigarette cravings appears to be comparable for both light and heavy smokers. Although an association between habitual PA and SOU was identified, an explanation for this link is beyond the scope of this study and requires further research. Speculatively, several mechanisms may be operating alone or in combination at different times for different people (Faulkner and Carless 2006). A new conceptual model for smoking cessation suggests that habitual PA may help to attenuate the smoking-induced decline in executive functioning impairment (Loprinzi et al. 2015a). The same authors found statistically significant associations between habitual PA and psychological outcomes in two large cross-sectional studies; smokers who were less physically active were also more likely to be depressed (Loprinzi et al. 2014), and exercise was found to facilitate smoking cessation via exercise-induced increases in smoking-specific self-efficacy in young smokers (Loprinzi et al. 2015b).

Finally, alcohol consumption was found to moderate the relationship between moderate intensity PA and SOU. The interactions revealed that for ‘heavy drinkers’, cravings increased as PA increased. It appeared that smokers consuming seven or more drinks per day on a typical day they drink lost the protective effect of habitual PA on SOU. For reasons which are unclear, this finding suggests that not all smokers may benefit from increasing their PA levels; however, more research is needed to replicate this effect.

Based on the results of the current study, further research exploring the association between habitual PA and cigarette cravings and other smoking cessation outcomes is needed before a role of habitual PA in smoking cessation can be established. Similarly, an investigation into the moderating effects of alcohol drinking is warranted. Habitual PA and alcohol consumption could be recommended as a routine measure in new smoking cessation trials. This would help to determine the role of PA and alcohol consumption in smoking cessation.

### Limitations

All results presented and discussed in this paper are based on exploratory post hoc cross-sectional analyses; therefore, the direction of any associations cannot be determined. The results seems to either suggest that doing more PA will lead to lower cigarette cravings or smokers with lower cravings are likely to do more PA. However, the findings might be very different for abstinent smokers or smokers trying to quit. In addition, the analyses here relied on self-reported PA and cigarette cravings data as reported over the last week; more objective measures such as accelerometry PA data may be needed. Also, it may be that PA data collection over 7 days is not sufficiently long to measure the chronic level of PA. Similarly, multiple SOU measures over a longer period of time may be needed to assess the level of trait cravings. Finally, alcohol consumption, the moderator of the relationship between moderate intensity PA and SOU, consists only of the frequency of alcohol consumption and pattern of drinking on a typical day. This approach has many limitations including having no indication of the total amount of alcohol consumed (which can be linked to safe consumption guidelines). However, there is a general lack of a standard classification for alcohol consumption (Furtwaengler and de Visser 2013; Nutt and Rehm 2014). In summary, all results must be interpreted with caution and should be only used as an indication for future research.

## Conclusion

Habitual PA was found to be significantly associated with SOU, with moderate PA being the best predictor. An increase in moderate PA was associated with lower SOU in disadvantaged smokers not ready to quit. Additional predictors of the effects of PA on SOU were identified; a higher MPSS score was associated with higher SOU, while a higher alcohol consumption was associated with a lower SOU. In addition, alcohol consumption was found to be a moderator of the relationship; for ‘heavy drinkers’ (defined as consuming seven or more alcoholic drinks on a typical day), increased minutes of moderate PA appeared to be associated with higher cravings. However, results of this study are exploratory and must be interpreted with caution. Further research into the moderating effects of alcohol drinking on the effects of habitual PA on cigarette cravings is needed.

## Electronic supplementary material

Below is the link to the electronic supplementary material.

ESM 1  (DOC 30 kb)

ESM 2  (DOC 33 kb)

ESM 3  (DOC 35 kb)
