# Supplementary material for: The association between habitual physical activity and cigarette cravings, and influence of smokers’ characteristics in disadvantaged smokers not ready to quit
Source: Psychopharmacology (Berl). 2016 Jun 2;233:2765–74. doi: 10.1007/s00213-016-4326-6 (PMC4917568; doi:10.1007/s00213-016-4326-6)
Supplement: Supplementary file 1 — (DOC 30 kb) [file 213_2016_4326_MOESM1_ESM.doc]

### Electronic Supplementary Material

#### Online Resource 1

Participant characteristics; alcohol variables

| **Variables** | **Mean (SD),**  **Median [IQR]** | **Proportions, (%; n/N):** |
| --- | --- | --- |
| How often do you have a drink containing alcohol? (N = 99) | 2.9 (1.2);  3 [2, 4] | Never: 15 (15/99)  Once a month or less: 26 (26/99)  2−4 times a month: 26 (26/99)  2−3 times a week: 20 (20/99)  4 times a week or more: 12 (12/99) |
| How many drinks containing alcohol do you have on a typical day when you are drinking? (N = 84) | 2.6 (1.3);  2 [1, 4] | 1 or 2 drinks: 27 (23/84)  3 or 4 drinks: 24 (20/84)  5 or 6 drinks: 20 (17/84)  7 to 9 drinks: 18 (15/84)  10 or more drinks: 11 (9/84) |
| How many drinks containing alcohol have you had in the past week? (N = 84) | 3.4 (2.1);  3 [1, 6] | None: 30 (25/84)  1 or 2 drinks: 7 (14/84)  3 or 4 drinks: 8 (7/84)  5 or 6 drinks: 7 (6/84)  7 to 9 drinks: 7 (6/84)  10 or more drinks: 31 (26/84) |

Key: N; Number of participants; SD; standard deviation; IQR; inter-quartile range.
