# Supplementary material for: The association between habitual physical activity and cigarette cravings, and influence of smokers’ characteristics in disadvantaged smokers not ready to quit
Source: Psychopharmacology (Berl). 2016 Jun 2;233:2765–74. doi: 10.1007/s00213-016-4326-6 (PMC4917568; doi:10.1007/s00213-016-4326-6)
Supplement: Supplementary file 2 — (DOC 33 kb) [file 213_2016_4326_MOESM2_ESM.doc]

#### Online Resource 2

Spearman correlations between background variables and Strength of Urge (N = 99)

| **Measure** | **Correlation coefficient** | **P-value** |
| --- | --- | --- |
| EQ-5D-3L | -0.23 | 0.024 |
| PSS | 0.19 | 0.064 |
| MPSS^a^ | 0.33 | 0.001 |
| mCEQ satisfaction^a^ | 0.11 | 0.293 |
| mCEQ reward^a^ | 0.29 | 0.004 |
| Cigarettes smoked per day | 0.08 | 0.415 |
| FTCD^a^ | 0.32 | 0.001 |
| Age when participants started smoking (years) | -0.13 | 0.194 |
| “*Alcohol drinking frequency*” | -0.23 | 0.022 |
| “*Drinks on a typical day*” ^b^ | 0.29 | 0.007 |
| “*Drinks in the past week*”) ^b^ | 0.13 | 0.233 |

Notes: a, Pearson correlation;; b, N = 84.

Key: EQ-5D-3L, three level European Quality of Life-5 Dimension questionnaire; MPSS, Mood and Physical Symptoms Scale; N, Number of participants; PSS, Perceived Stress Scale; FTCD, Fagerström Test for Cigarette Dependence; mCEQ, modified Cigarette Evaluation Questionnaire.
