# Supplementary material for: The association between habitual physical activity and cigarette cravings, and influence of smokers’ characteristics in disadvantaged smokers not ready to quit
Source: Psychopharmacology (Berl). 2016 Jun 2;233:2765–74. doi: 10.1007/s00213-016-4326-6 (PMC4917568; doi:10.1007/s00213-016-4326-6)
Supplement: Supplementary file 3 — (DOC 35 kb) [file 213_2016_4326_MOESM3_ESM.doc]

#### Online Resource 3

Series of linear regression models investigating the effects of each potential additional predictor individually on Strength of Urge (not adjusted for moderate intensity physical activity, N = 99)

|  | | **Strength of Urge** | |
| --- | --- | --- | --- |
| ***Predictors*** | | **Mean difference**  **(95% CI)** | **t statistics**  **(p)** |
| *EQ-5D-3L* | | -14.99  (-31.86; 1.88) | -1.76  (0.081) |
| *PSS* | | **1.21**  **(0.10; 2.33)** | **2.16**  **(0.033)** |
| *MPSS* | | **8.72**  **(3.67; 13.78)** | **3.43**  **(0.001)** |
| *FTCD* | | **3.65**  **(1.46; 5.85)** | **3.31**  **(0.001)** |
| *mCEQ reward* | | **5.43**  **(1.75; 9.12)** | **2.93**  **(0.004)** |
| *“Alcohol consumption”^a, b^* | *Light*  *drinkers* | **-26.67**  **(-39.28; -10.88)** | Global statistic^c^:  **F_(2,96)_ = 11.54, p < 0.001** |
|  | *Heavy drinkers* | **-11.17**  **(-31.58; -3.19)** |  |
| *Employment status* | | 8.52  (-0.75; 17.79) | 1.82  (0.071) |
| *Presence of a mental health condition^d^* | | **12.56**  **(3.37; 21.75)** | **2.71**  **(0.008)** |
| *Met PA guidelines^b^* | | -5.86  (-16.07; 4.34) | -1.14  (0.257) |
| *Gender* | | 3.48  (-5.97; 12.93) | 0.73  (0.466) |

Notes: a, “non-drinkers” (no alcohol consumption), “light drinkers” (consuming between 1 and 6 alcoholic drinks on a typical day), and “heavy drinkers” (consumed 7 or more alcoholic drinks on a typical day); b, N = 98; c, = p-values for the global F statistic of the three levels alcohol consumption are derived from a Wald test; d, answered ‘moderately’ or ‘extremely’ anxious or depressed to item 5 of the EQ-5D-3L questionnaire; “non-drinkers” was the baseline category for drinking alcohol; “male” was the baseline category for gender; “employed” was the baseline category for employment status; “not meeting PA guidelines” was the baseline category for Met PA guidelines; ”lack of anxiety” was the baseline category for anxiety present.

Key: BMI, body mass index (kg/m2); EQ-5D-3L, three level European Quality of Life-5 Dimension questionnaire; FTCD, Fagerström Test for Cigarette Dependence; mCEQ, modified Cigarette Evaluation Questionnaire; N, Number of participants; MPSS, Mood and Physical Symptoms Scale; PA, physical activity; PSS, Perceived Stress Scale.
